# Regulation of cell cycle transition and induction of apoptosis in HL-60 leukemia cells by lipoic acid: role in cancer prevention and therapy

**DOI:** 10.1186/1756-8722-1-4

**Published:** 2008-05-30

**Authors:** Elangovan Selvakumar, Tze-chen Hsieh

**Affiliations:** 1 Department of Biochemistry and Molecular Biology, New York Medical College, Valhalla, New York 10595, USA

## Abstract

**Background:**

Lipoic acid (LA), a potent antioxidant, has been used as a dietary supplement to prevent and treat many diseases, including stroke, diabetes, neurodegenerative and hepatic disorders. Recently, potent anti-tumorigenic effects induced by LA were also reported and evident as assayed by suppression of cell proliferation and induction of apoptosis in malignant cells. However, the mechanism by which LA elicits its chemopreventive effects remains unclear.

**Methods and Results:**

Herein, we investigated whether LA elicits its anti-tumor effects by inducing cell cycle arrest and cell death in human promyelocytic HL-60 cells. The results showed that LA inhibits both cell growth and viability in a time- and dose-dependent manner. Disruption of the G_1_/S and G_2_/M phases of cell cycle progression accompanied by the induction of apoptosis was also observed following LA treatment. Cell cycle arrest by LA was correlated with dose-dependent down regulation of Rb phosphorylation, likely via suppression of E2F-dependent cell cycle progression with an accompanying inhibition of cyclin E/cdk2 and cyclin B1/cdk1 levels. Evidence supporting the induction of apoptosis by LA was based on the appearance of sub-G_1 _peak in flow cytometry analysis and the cleavage of poly(ADP-ribose) polymerase (PARP) from its native 112-kDa form to the 89-kDa truncated product in immunoblot assays. Apoptosis elicited by LA was preceded by diminution in the expression of anti-apoptotic protein bcl-2 and increased expression of apoptogenic protein bax, and also the release and translocation of apoptosis inducing factor AIF and cytochrome c from the mitochondria to the nucleus, without altering the subcellular distribution of the caspases.

**Conclusion:**

This study provides evidence that LA induces multiple cell cycle checkpoint arrest and caspase-independent cell death in HL-60 cells, in support of its efficacious potential as a chemopreventive agent.

## Background

α-Lipoic acid (LA), also known as thioctic acid, occurs naturally as a prosthetic group in various mitochondrial enzymatic complexes and plays a fundamental role in metabolism. It is involved in different multienzyme complexes such as pyruvate dehydrogenase, α-ketoglutarate dehydrogenase, branched-chain α-keto acid dehydrogenase, and glycine decarboxylase complex [1]. The two sulfur molecules in LA undergo cycles of oxidation and reduction, enabling it to function as a potent antioxidant that is capable of directly terminating potentially damaging free radicals. Several features have been described for LA such as (a) specificity of free radical scavenging in both oxidized and reduced forms, (b) interaction with other antioxidants, (c) metal-chelating activity, (d) effects on gene expression, (e) bioavailability, (f) location (in aqueous or membrane domains, or both), and (g) ability to repair oxidative damage, which make it an outstanding antioxidant [2-4]. Added to cell culture medium *in vitro*, LA readily enters cells and is reduced by mitochondrial and cytosolic enzymes to dihydrolipoic acid, most of which is rapidly effluxed from the cell to the culture medium [5]. Experimental and clinical studies have indicated the potential usefulness of exogenous LA as a therapeutic agent for the prevention and treatment of various pathologies including diabetes [6], atherosclerosis [7], ischemia-reperfusion injury [8], degenerative processes in neurons [9], diseases of joints [10], radiation injury [11], heavy metal poisoning [12] and HIV activation [13]. LA is readily absorbed from the diet, and to date, only mild side effects have been detected following LA administration; supports the overall feasibility of using LA as a dietary supplement [3].

In recent years, LA has gained considerable attention in the cancer field as an anticancer agent [14,15]. Results from antiproliferation studies on cancerous cell-based models have suggested that the tumor-suppressive effect of LA corresponds with apoptosis induction, a critical parameter impaired in cancer cells, and this induction is selectively exerted in cancer and transformed cell lines, while being less active toward normal nontransformed cells [16-18]. Thus, LA was shown to induce apoptosis in tumor Jurkat, FaDu, Ki-v-Ras-transformed mesenchymal cells and human lung epithelial cancer H460 cells [19,20]. In human leukemic T cells, LA also potentiated Fas-mediated apoptosis through redox regulation without affecting peripheral blood monocytes from healthy humans [21]. In experiments using antioxidant response element (ARE) reporter assays, LA has also been shown to induce phase II protective genes which are involved in the prevention of carcinogenesis, in non-cancerous animal- and cell-based studies [22-24]. These studies support the potential utility of LA as an anticancer agent and the importance of the elucidation of the detailed mechanism of its antitumor activity. Because of its widespread use and therapeutic potential of LA, however, the mechanism by which LA elicits its chemopreventive effects remains largely unknown.

We sought to determine the LA-induced apoptosis and cell cycle arrest and the underlying mechanisms of action. Our study shows for the first time that LA is capable to block multiple cell cycle checkpoints including G_1_/S and G_2_/M and induce caspase-independent cell death via AIF/cytochrome c translocation from the mitochondria to the nucleus. Our findings provide mechanistic support to the potential utility of LA as an agent for the treatment of leukemia.

## Materials and methods

### Reagents

DL-α-Lipoic acid was purchased from LKT laboratories (St Paul, MN). Primary antibodies like anti-Rb, anti-E2F, anti-cyclin B1, anti-cyclin D, anti-cyclin E, anti-cdk1, anti-cdk2, anti-AIF, anti-cytochrome c, anti-bcl-2, anti-bax, anti-actin, anti-histone H1, and secondary antibodies were purchased from Santa Cruz Biotechnology, Inc. (Santa Cruz, CA). Primary antibodies like anti-pRb (ser 780) and anti-pRb (ser 807/811) were purchased from Biosource International, Inc. (Camarillo, CA). Anti-PARP was purchased from Biomol International, L.P. (Plymouth Meeting, PA). Fetal calf serum, RPMI 1640, penicillin and streptomycin were purchased from Cellgro, Inc (Herndon, VA). All other chemicals and solvents used were of analytical grade.

### Cell culture and growth inhibition assay

Human HL-60 cells were obtained from American Tissue Culture Collection (Manassas, VA) and maintained in RPMI 1640 supplemented with penicillin, streptomycin and 10% heat inactivated fetal calf serum as previously described [25-27]. For treatment, cells were seeded at a density of 1 × 10^5 ^cells/ml. LA dissolved in 1 N NaOH solution and neutralized with HCl, was added to the culture media to the final concentration specified in the text. At the specified times, control and treated cells were harvested. Cell count was performed using a hemocytometer and cell viability was determined by trypan blue exclusion [25-27]. Harvested cells were washed twice with PBS, and pellets were stored at -80°C for additional biochemical and molecular analyses.

### Cell cycle analysis

Cell cycle phase distribution was assayed by flow cytometry. Following 24 and 48 h treatment of HL-60 cells with different concentrations of LA (0, 2.5, and 5 mM), cells were washed with PBS and stained with 1.0 μg/ml DAPI containing 100 mM NaCl, 2 mM MgCl_2 _and 0.1% Triton X-100 (Sigma) at pH 6.8, as described [26,28,29]. The DNA-specific DAPI fluorescence was excited with UV light emitting laser (Ni-Cad), and collected with appropriate filters in an ICP-22 (Ortho Diagnostic, Westwood, MA) flow cytometer. MultiCycle software from Phoenix Flow Systems (San Diego, CA) was used to deconvolute the cellular DNA content histograms to obtain quantitation of the percentage of cells in the respective phases (G_1_, S and G_2_/M) of the cell cycle. Flow cytometry was also used to show cells undergoing apoptosis, evident by the appearance of the sub-G_1 _peak [26,28,29].

### Preparation of whole cell extracts and subcellular fractionation

For immunoblotting experiments, cells were collected by centrifugation and were lysed in ice-cold RIPA buffer (50 mM Tris, pH 7.4, 150 mM NaCl, 1 mM EDTA, 1% Triton X-100, 1% deoxycholate, 0.1 % SDS, 1 mM dithiothreitol and 10 μl/ml protease inhibitor cocktail). The extracts were centrifuged and the clear supernatants were stored in aliquots at -70°C for further analysis. Subcellular fractionation was performed using mitochondria isolation kit obtained from Sigma (Sigma Chemicals, St Louis, MO) and different compartmental proteins were used to study the translocation of AIF and cytochrome c. Protein content of cell lysates and subcellular fractions was determined by coomassie protein assay kit (Pierce, Rockford, IL) with BSA as standard.

### Immunoblotting

The aliquots of lysates (20 μg of protein) were boiled with sample buffer for 5 min, and resolved by 10% SDS-PAGE. The proteins were transferred to a nitrocellulose membrane and blocked in TBST buffer (10 mM Tris, pH 7.5, 100 mM NaCl and 0.05% Tween 20) containing 3% nonfat dried milk overnight at 4°C. The blots were incubated with various primary antibodies, followed by incubation for 1 h with appropriate secondary antibodies conjugated to horseradish peroxidase in TBST. Actin and histone expression was used as loading control. Fractionation of the mitochondrial and nuclear proteins was confirmed by probing the membrane for mitochondrial specific cytochrome c oxidase antibody or nuclear specific histone H1 using their specific antibodies. The intensity of the specific immunoreactive bands were detected by enhanced chemiluminescence (ECL), using the manufacturer's protocol (Kirkegared & Perry Laboratories) and quantified by densitometry and expressed as a ratio to actin or histone, as previously described [27].

## Results

### Inhibition of HL-60 cell growth by LA is both time and dose dependent

**Figure 1:**
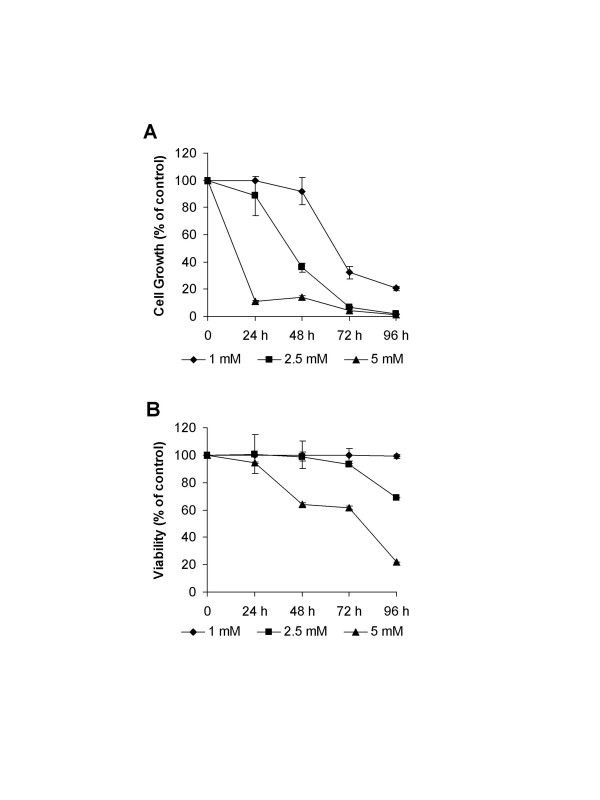
Control of cell growth and viability in HL-60 cells by LA. (A) Cells were treated with 0, 1, 2.5 and 5 mM LA and the cell numbers were determined at 24, 48, 72 and 96 h. (B) Cell viability was measured using the trypan blue dye exclusion assay. Effects of LA were presented as a percentage of control, and values are expressed as mean ± SD for three experiments.

Initially, we investigated the effect of LA on cell growth inhibition. Exponentially growing HL-60 cells were treated with increasing doses and exposure times of LA, and subjected to trypan blue exclusion assay to measure the cell growth and viability. LA treatment resulted in dose- and time-dependent inhibition of cell growth, compared with controls, and the magnitude of cell growth suppression was seen as early as 24 h exposure to 5 mM LA (89%; Fig. 1A). By 48 h there was a ~8%, ~64% and 86% diminution of cell growth by 1, 2.5 and 5 mM LA, respectively, which was accompanied by ~1, ~3% and 36% temporal, dose-dependent decrease in cell viability (Fig. 1B).

### LA induces HL-60 cell cycle arrest by altering the expressions of specific signaling proteins

**Figure 2:**
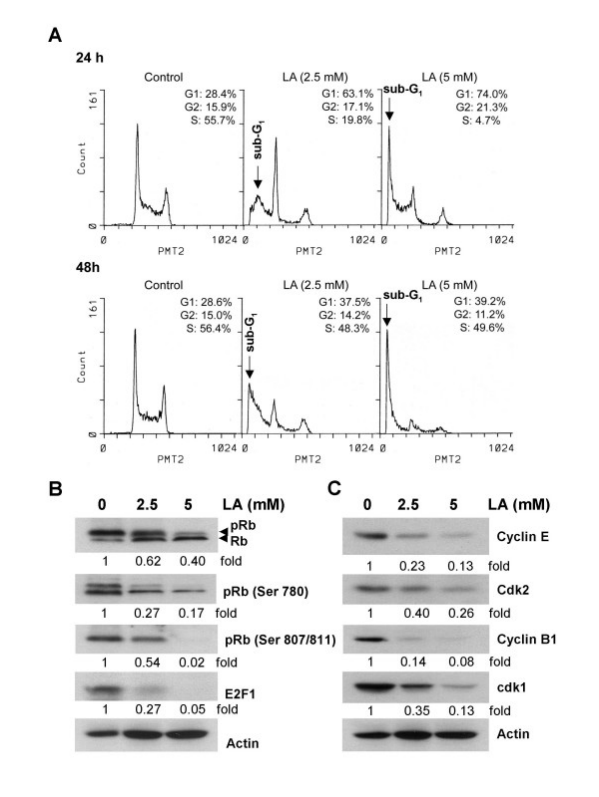
Effects of LA on cell cycle phase distribution and the expression of various cell cycle regulatory proteins in HL-60 cells. (A) Cells were treated with 0, 2.5 and 5 mM LA for 24 and 48 h and analyzed by flow cytometry. Cells with hypodiploid DNA content (sub-G_1_) represent apoptotic cell fractions. (B) Western blot analysis of total Rb, pRB (ser780), pRB (ser 807/811) and E2F expression in cell lysate treated with LA for 48 h. (C) The level of immunoreactive cyclins B1, E, cdk1 and cdk2 in LA-treated HL-60. The intensity of the specific immunoreactive bands were quantified by densitometry and expressed as a fold difference against actin.

To assess LA-induced cell growth suppression is mediated via alterations in cell cycle, we evaluated the cell cycle distribution by flow cytometry. Since 48 h treatment with 1 mM LA showed minimum affects on cell growth and viability, only cells exposed to 2.5 and 5 mM LA for 24 and 48 h were analyzed. The percentage of cells in G_1_, S, and G_2 _phases were calculated and presented as histograms in Fig. 2A. LA caused a significant decrease in S-phase cell population (55.6% in control vs. 19.8% and 4.7% in cells treated with 2.5 and 5 mM LA, respectively), accompanied by a concomitant accumulation in the G_1 _phase cell population (28.4% in control vs. 63.1% and 74% in 2.5 and 5 mM LA treated cells). To further explore the cell cycle arrest by LA in HL-60 cells, specific cell cycle regulatory proteins required for G_1_, G_1_/S and S phase transition were measured by Westerm blot analysis. First, we measured the expressions of cyclins D, E and cdk2, as they play a pivotal role in controlling the phosphorylation status of Rb, which in turn activate transcription factor E2F to induce cell entry into the S-phase. Results in Fig. 2B show that LA treatment caused a dose-dependent reduction in cyclin E/cdk2 expression without affecting cyclin D1 (data not shown), and at the same time LA treatment also resulted in ~38 to 60% suppression of the phosphorylated Rb (pRb). Moreover, LA caused a significant reduction in the phosphrylation of Rb at two specific sites, Ser-780 and Ser-807/811, was also observed (Fig. 2B). In addition, a more pronounced decrease in the expression of E2F was also detected in the treated cells (Fig. 2B), suggesting that these changes collectively contributed to the decrease in S phase cell population by LA (Fig. 2A).

Since LA-treated cells also show alterations in G_2_/M progression, we also assayed the expression of cyclins A, B and cdk1 expression and observed a dose-dependent down regulation of cyclin B1/cdk2 (Fig. 2C) without a corresponding alteration in the expression of cyclin A (data not shown).

### LA induces apoptosis by increasing bax/bcl2 ratio and by causing poly(ADP-ribose) polymerase (PARP) cleavage

**Figure 3:**
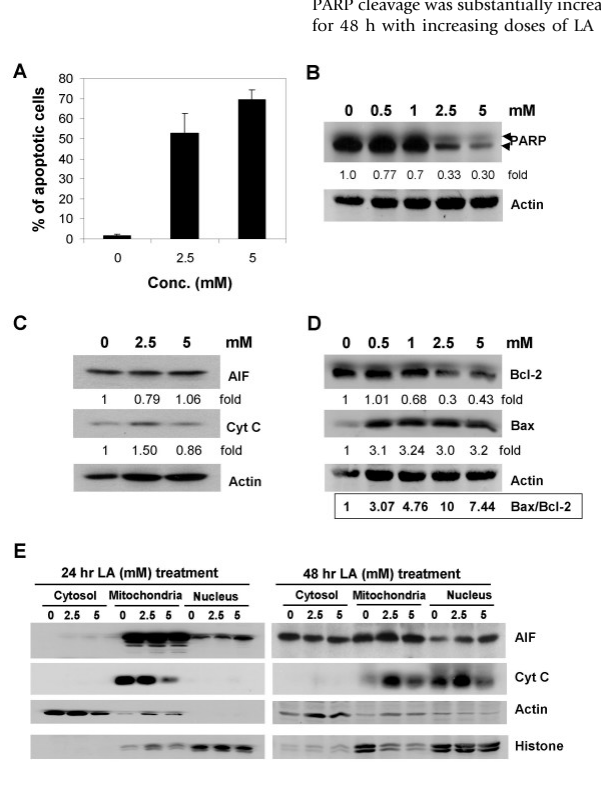
Induction of apoptosis by LA and analysis on poly(ADP-ribose) polymerase (PARP) cleavage, AIF/cytochrome c expression, and bax/bcl-2 ratio and subcellular distribution of AIF/cytochrome c by LA. (A) HL-60 cells were treated with 0, 2.5 and 5 mM LA for 24 to 48 h; LA induced cell death, evident by the flow cytometric measured sub-G1 fraction was calculated and shown as % of total cell population. (B) Western blot analysis revealed down regulation of PARP expression at accompanied by appearance of 89 kDa cleaved PARP fragment in ≥ 2.5 mM, 48 h LA treated cells. (C) AIF and cytochrome c (Cyt C) expression in 48 h LA treated cells. (D) The actin-adjusted level of bax and bcl-2 and changes in the ratio of bax to bcl-2 in HL-60 cells treated for 48 h with increasing dose of LA. (E) Subcellular distribution of immunoreactive AIF and Cyt C in the cytosol, mitochondria and nucleus in control and 24 and 48 h LA-treated HL-60 cells. Actin and histone was used as loading control for cytosol and nucleus fractions, respectively. For mitochondria fraction verification was performed as detailed in Methods.

Cell cycle analysis revealed that LA apparently induced apoptosis as evident by the appearance of sub-G_1 _fraction (Fig. 2A); notably, the percentage of apoptotic cells increased from 1.4% in control cells to 59.6% and 72.9% in 24 and 48 h, 2.5 and 5 mM LA-treated cells, which might contribute to the growth inhibitory effects of LA (Fig. 3A). Corroborative evidence of induction of apoptosis was obtained by biochemical analysis showing that PARP cleavage was substantially increased in cells treated for 48 h with increasing doses of LA (Fig. 3B). As additional support, other apoptosis markers including AIF, cytochrome c and bax/bcl-2 ratio were also examined to further ascertain the response of cells to LA treatment, by western blot analysis. Treatment of HL-60 cells with 2.5 mM LA for 24 h resulted in a 1.5 fold increase in total cytochrome c, while the total AIF levels remained unchanged (Fig. 3C). As bcl-2 plays an integral role in the release of cytochrome c during cell death, we determined its expression and correspondingly, also bax, an apoptosis agonist, in control and LA-treated whole cell extracts. Western blot analysis clearly showed a dose-dependent suppression of bcl-2 expression, accompanied by concomitant increases in bax, in LA-treated cells, compared to control cells (Fig. 3D), which was most vividly illustrated as a marked increase in bax-to-bcl-2 expression ratio (Fig. 3D). These results further support the ability of LA to activate the mitochondria-dependent apoptotic cascade.

### LA induces translocation of cytochrome c and AIF

Induction of apoptosis by LA conceivably may involve the translocation of cytochrome c and AIF. This possibility was tested by biochemically fractionating different subcellular compartments and quantifying the appearance of cytochrome c and AIF by western blot analysis, following treatment with LA. Typical results in cells treated with 2.5 and 5 mM LA for 24 and 48 h showed a spatiotemporal release of AIF from mitochondria into the nucleus (Fig. 3E). Similarly, cytochrome c was also apparently released from the mitochondria, and unexpectedly, was not accompanied by a concomitant cytoplasmic increase (Fig. 3E). These results suggest that LA-elicited cell death may not occur via a classical cytochrome c mitochondria-cytosol translocation mechanism but rather, a caspase-independent mode of cell death via the nucleus directed shuttling of AIF and cytochrome c.

## Discussion

**Figure 4:**
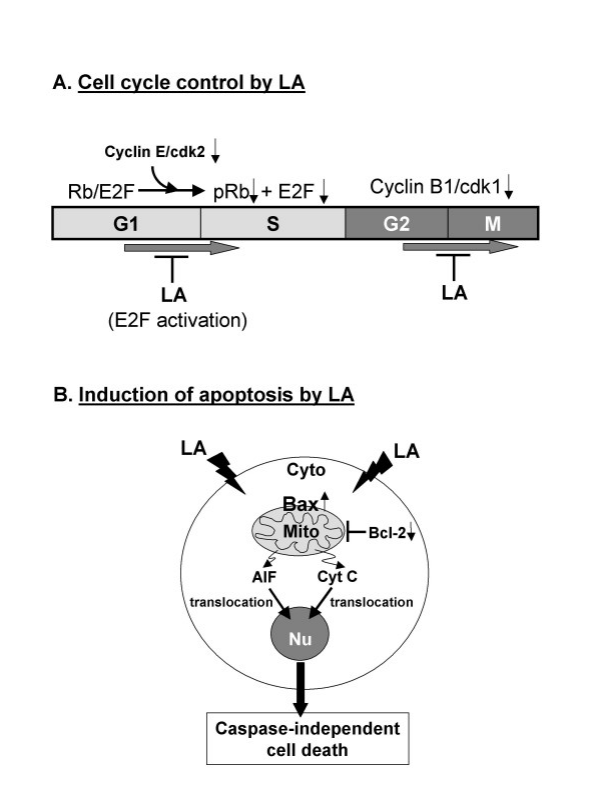
Proposed mechanism of action of LA. In this model, the ability of LA to suppress cell proliferation and induce apoptosis in HL-60 cells is hypothesized to involve (A) disruption of cell cycle control, (B) perturbation in apoptogenic/anti-apoptotic (bax/bcl-2) regulatory protein expression and translocation of mitochondrial AIF and cytochrome c (Cyt C) from mitochondria to nucleus and promoting the caspase-independent induction of apoptosis.

LA has pleiotropic pharmacologic effects. The therapeutic potential of LA in cancer treatment has been shown in several studies [14,17,20], however, the mechanisms by which LA elicits its chemopreventive properties remain largely unknown. Using HL-60 cells, we have confirmed the cancer cell growth suppressive effects of LA. Further, we now provide evidence for two novel LA-elicited changes that possibly contribute to its chemopreventive potentials: (i) LA induces blockade at both well established cell cycle checkpoint, respectively, G_1_/S and G_2_/M, (ii) LA promotes the demise of treated HL-60 cells, possibly by a combination of mechanisms that includes the mitochondria-dependent apoptotic cascade encompassing a caspase-independent mode of cell death mediated via the translocation of AIF/cytochrome c. The proposed mechanism of LA is depicted in Figure 4.

Targeting dual checkpoints of the cell cycle by LA is particularly noteworthy as it effectively, as a single agent, accomplishes the same cellular endpoint as what has been eloquently proposed by Li et al [30] of inducing malignant cell demise through the deliberate bi-checkpoint blockade-mediated induction of apoptosis, as exemplified by the combined administration of β-lapachone and taxol to deliver a one-two punch for tumor cell killing and eradication. The mechanism by which LA acts in dual cell cycle checkpoint control may be complex and appears to involve at least the down regulation of cyclin E/cdk2 and cyclin B1/cdk1 in a manner that effects synergistic cell cycle arrest and induction of apoptosis [30,31]. It is notable that earlier studies have also demonstrated the post-translational elevation of p27Kip1 and p21Cip1 as specific LA elicited effects [14,19]. Taken together, these results not only reinforce the essential role of LA in cell cycle control but are likely to be directly involved in contributing to its therapeutic potential in cancer treatment.

Results of flow cytometry analysis assessing the presence of cells with fractional DNA content (evident as the sub-G_1 _peak), in combination with the appearance of specifically processed 89-kD PARP product as demonstrated by immunoblot analysis (Figures 2 and 3), showed clearly the restoration/activation of programmed cell death in HL-60 cells treated with LA. Since the flow cytometric data appeared to show a more pronounced effect of prolonged treatment by LA, especially at the higher concentrations it is possible that more than one mode of cell death is triggered by LA. Equally likely is the possibility that these two assays alone are not sufficiently definitive to establish the mode of cell death in the treated cells. Experiments exploring TUNEL and agarose gel electrophoresis for detecting appearance of DNA ladders, and the use of caspase inhibitors are contemplated to address these and other possibilities. Despite the limitations mentioned above, it is important to point out a significant finding in this study, i.e., the demonstration of translocation of two proteins, respectively, AIF and cytochrome c from mitochondria to the nucleus after LA treatment. A dose-dependent increase of AIF appearing in the nuclear fraction was observed as early as 24 h, whereas cytochrome c release and nuclear accumulation occurred at 48 h. Recent studies have demonstrated that AIF plays a critical role in caspase-independent induction of apoptosis [32,33]. Our studies also showed that LA down regulated bcl-2 expression, which in turn may aid the release of AIF by altering mitochondrial permeability and contributing to its relocalization to the nucleus, and thereby promoting the induction of caspase-independent apoptosis. A companion and equally important change in this regard may be the cellular fate of cytochrome c, which, in our studies of the effects of LA, became nuclear bound. It is notable that previous studies have demonstrated a novel role of cytochrome c in the activation caspase-independent apoptosis, as involving the nucleus accumulation of cytochrome c instead of a more generally accepted classical mechanism in which the cytoplasmic translocation of cytochrome c from mitochondria provides a key trigger for caspase-dependent apoptosis [34]. Indeed, there is increasing awareness and acceptance regarding the co-existence of caspase-dependent and caspase-independent apoptotic and other modes of cell death for a given cell type [35]. Such as notion is consistent with and supported by our observation of the re-localization of mitochondrial proteins, AIF and cytochrome c into nucleus by LA treatment, suggesting that LA signals cell death in responsive cells by a caspase-independent, nuclear activated other apoptotic and perhaps other cell death mechanism.

Importantly, the concentrations of LA used in this study are similar to those used in other *in vitro *studies reporting the cell cycle arrest and apoptosis inducing properties of LA [19]. Notably also, mM LA concentrations have been reported in the plasma after oral dosing in pharmacokinetic studies and are considered non-toxic. Moreover, the half-life of LA in plasma is short (30 min), suggesting that it is rapidly taken up into tissues or further metabolized [36]. Therefore, it is plausible that high concentrations, in the mM range, may accumulate in target tissues.

## Conclusion

The results of this study demonstrate conclusively that LA treatment causes cell cycle arrest and alterations in the expression/translocation of mitochondrial apoptogenic/anti-apoptotic proteins including AIF and cytochrome c, and the net result being a reduction in cell proliferation concomitant with cell cycle arrest and induction of apoptosis. These findings may be part of the mechanisms that underlie or contribute to the beneficial effects of this readily available dietary supplement in cancer prevention.

## Authors' contributions

ES carried out the studies in Figure 1, Figure 2B and 2C, and Figure 3B–E, TCH carried out the studies in Figure 2A, Figure 3A and Figure 4, TCH conceived of the study, and participated in its design and coordination. All authors read and approved the final manuscript.
